# Bis(2-amino-3-nitro­pyridinium) dihydrogen­diphosphate

**DOI:** 10.1107/S1600536810000942

**Published:** 2010-01-16

**Authors:** Samah Toumi Akriche, Mohamed Rzaigui, Zeid Abdellah Elothman, Refaat Mohamed Mahfouz

**Affiliations:** a Laboratoire de Chimie des Matériaux, Faculté des Sciences de Bizerte, 7021 Zarzouna Bizerte, Tunisia; b Chemistry Department, Faculty of Science, King Saud University, PO Box 2455, Riyadh 11451, Saudi Arabia

## Abstract

The structure of the title compound, 2C_5_H_6_N_3_O_2_^+^·H_2_P_2_O_7_^2−^, contains infinite (H_2_P_2_O_7_^2−^)_*n*_ layers stacked perpendicular to the *a* axis. The 2-amino-3-nitro­pyridinium cations are arranged in pairs and are anchored between these layers, linking them by N—H⋯O and C—H⋯O hydrogen-bonding and electrostatic inter­actions between anionic and cationic species to form a three-dimensional network.

## Related literature

For related structures of 2-amino-3-nitro­pyridinium, see: Akriche & Rzaigui (2000 ▶, 2009*a* ▶,*b* ▶,*c* ▶); Nicoud *et al.* (1997 ▶). For bond lengths in related structures, see: Aakeröy *et al.* (1998 ▶). For related structures of diphosphate anions, see: Akriche & Rzaigui (2005 ▶); Charfi & Jouini (2005 ▶); Brodski *et al.* (2004 ▶); Mrad *et al.* (2006 ▶); Soumhi *et al.* (1998 ▶).
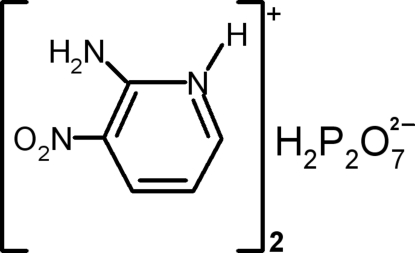


## Experimental

### 

#### Crystal data

2C_5_H_6_N_3_O_2_^+^·H_2_O_7_P_2_^2−^
*M* *_r_* = 456.21
Orthorhombic, 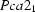

*a* = 34.250 (5) Å
*b* = 5.763 (2) Å
*c* = 8.991 (3) Å
*V* = 1774.8 (9) Å^3^
*Z* = 4
Mo *K*α radiation
μ = 0.32 mm^−1^
*T* = 298 K
0.29 × 0.25 × 0.19 mm

#### Data collection

Enraf–Nonius CAD-4 diffractometer
2726 measured reflections
2724 independent reflections
2279 reflections with *I* > 2σ(*I*)
*R*_int_ = 0.008
2 standard reflections every 120 min  intensity decay: 3%

#### Refinement

*R*[*F* ^2^ > 2σ(*F* ^2^)] = 0.033
*wR*(*F* ^2^) = 0.091
*S* = 1.09
2724 reflections
265 parameters
1 restraint
H-atom parameters constrained
Δρ_max_ = 0.53 e Å^−3^
Δρ_min_ = −0.28 e Å^−3^
Absolute structure: Flack (1983 ▶), 443 Friedel pairs
Flack parameter: −0.15 (11)

### 

Data collection: *CAD-4 EXPRESS* (Enraf–Nonius, 1994 ▶); cell refinement: *CAD-4 EXPRESS*; data reduction: *XCAD4* (Harms & Wocadlo, 1995 ▶); program(s) used to solve structure: *SHELXS97* (Sheldrick, 2008 ▶); program(s) used to refine structure: *SHELXL97* (Sheldrick, 2008 ▶); molecular graphics: *ORTEP-3 for Windows* (Farrugia, 1997 ▶) and *DIAMOND* (Brandenburg & Putz, 2005 ▶); software used to prepare material for publication: *WinGX* (Farrugia, 1999 ▶).

## Supplementary Material

Crystal structure: contains datablocks I, global. DOI: 10.1107/S1600536810000942/dn2529sup1.cif

Structure factors: contains datablocks I. DOI: 10.1107/S1600536810000942/dn2529Isup2.hkl

Additional supplementary materials:  crystallographic information; 3D view; checkCIF report

## Figures and Tables

**Table 1:** Hydrogen-bond geometry (Å, °)

*D*—H⋯*A*	*D*—H	H⋯*A*	*D*⋯*A*	*D*—H⋯*A*
O2—H2⋯O5^i^	0.82	1.73	2.549 (3)	178
O7—H7⋯O1^ii^	0.82	1.73	2.537 (3)	166
N1—H1⋯O1	0.86	1.87	2.706 (3)	165
N2—H2*A*⋯O3	0.86	1.88	2.726 (4)	168
N2—H2*B*⋯O9	0.86	2.05	2.645 (4)	126
N4—H4⋯O6	0.86	1.72	2.582 (4)	174
N5—H5*A*⋯O5	0.86	1.95	2.786 (4)	165
N5—H5*B*⋯O10	0.86	2.07	2.669 (5)	126
N5—H5*B*⋯O3^iii^	0.86	2.22	2.843 (4)	130
C2—H2*C*⋯O7^iv^	0.93	2.46	3.280 (4)	147
C3—H3⋯O6^iv^	0.93	2.34	3.208 (4)	155
C8—H8⋯O9^v^	0.93	2.52	3.097 (4)	120

Symmetry codes: (i) 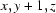; (ii) 
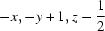
; (iii) 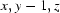; (iv) 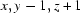; (v) 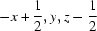.

**Fig. 1.**
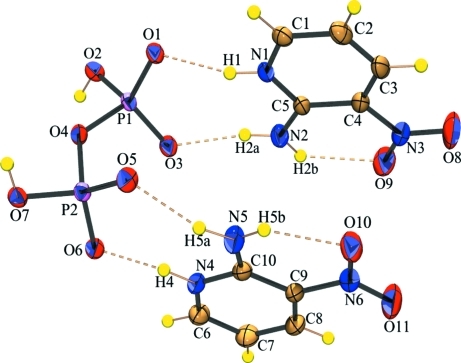
An ORTEP view of (I) with the atom-labelling scheme. Displacement ellipsoids are drawn at the 30% probability level. H atoms are represented as small spheres of arbitrary radii. Hydrogen bonds are represented as dashed lines.

**Fig. 2.**
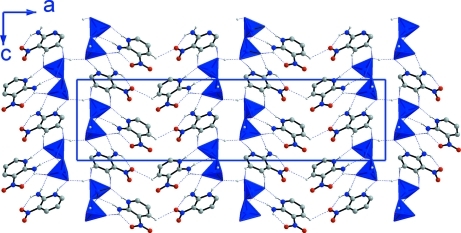
Projection of (I) along the b axis. The H-atoms not involved in H-bonding are omitted.

## supplementary crystallographic information

### Comment

In the framework of our systematic research using the 2-amino-3-nitropyridine (2 A3NP) molecule, we report here the new non-centrosymetric compound, 2(C_5_H_6_N_3_O_2_)^+^, H_2_P_2_O_7_^2-^ (I) obtained by the interaction of the 2 A3NP molecule and diphosphoric acid.

The asymmetric unit of  the title compound is built up from one anion H_2_P_2_O_7_^2-^ and two (C_5_H_6_N_3_O_2_)^+^ cations as shown in Fig. 1.

The dihydrogendiphosphate anions are connected through strong hydrogen bonds characterized by relatively short distances (with distances O2···O5 = 2.549 (3) Å and O7···O1 = 2.537 (3) Å (Table 1)), to form corrugated layers parallel to *bc* plane at *x* = 0 and *x*= 1/2 (Fig. 2). Two crystallographically independent cations coexist in this structure. They are arranged in pairs and anchored onto both adjacent anionic layers *via* N—H···O and C—H···O hydrogen bonds to keep up the three-dimensionel network cohesion.

As expected, the H_2_P_2_O_7_^2-^ group with bent configuration shows its standard geometry, the longest bonds length P2–O4 = 1.592 (2) Å and P1–O4 = 1.613 (2) Å, correspond to the bridging oxygen atom, the intermediate ones, P1–O2 = 1.552 (2) Å and P2–O7 = 1.545 (2) Å, correspond to the P–OH bonding and the shortest ones spreading between 1.481 (2) Å and 1.515 (2) Å, correspond to the external oxygen atoms. The average values of the P–O distances and O–P–O angles are 1.536 Å and 109.3°. The P–P distance is 2.898 (1) Å and the P–O–P angle is close to 129.4 (1) °. All these distances and angles are similar to those commonly observed in others diphosphate anions (Akriche & Rzaigui, 2005; Charfi & Jouini, 2005; Brodski, *et al.*, 2004; Mrad, *et al.*, 2006). Despite the limited number of organic cation diphosphates (about twenty seven related structures of diphosphate anions), we can distinguish only one non-centrosymmetric structure (Soumhi, *et al.*, 1998) such as the title compound (I).

In this atomic arrangement, one can distinguish the inter-cation contact C8—H8···O9 (H8···O9 = 2.52 Å) which induces the aggregation of the two independent organic cations in pairs (2 A3NP^+^)_2_. This kind of arrangement is also observed in the related structure of 2-amino-3-nitropyridinium hydrogenselenate (Akriche & Rzaigui, 2009*b*). These pairs are located between the anionic layers to link them by manifesting different interactions (Fig. 2). The geometric features of organic cations are usual and comparable with values of other 2-amino-3-nitropyridinium compounds (Akriche & Rzaigui, 2000; Nicoud *et al.*,1997; Akriche & Rzaigui, 2009*a*, 2009*b*, 2009*c*). It is worth noticing, the C—NH_2_ (1.316 (4) Å) and C—NO_2_ (1.440 (4) and 1.454 (5) Å) distances in the 2 A3NP cations are respectively shortened and lengthened with respect to the C—NH_2_ (1.337 (4) Å) and C—NO_2_ (1.429 (4) Å) observed in the 2-amino-3-nitropyridine molecular crystal (Aakeröy, *et al.*, 1998). All the 2-amino-3-nitropyridinium cations encapsulated in various anionic subnetworks show the same changes in C—NH_2_ and C—NO_2_ distances, revealing a weak increase of pi bond character in C—NH_2_ and a decrease in C—NO_2_.

### Experimental

Single crystals of the title compound were prepared at room temperature by slow evaporation of a mixture of an aqueous solution (20 ml) of diphosphoric acid (5 mmol) and an ethanolic solution (10 ml) of 2-amino-3-nitropyridine (4 mmol, 354 mg). The diphosphoric acid was produced from Na_4_P_2_O_7_ by using a cation-exchange resin (Amberlite IR 120). The resulting solution was evaporated slowly at room temperature for several days until the formation of good quality of prismatic single crystals.

### Refinement

All H atoms attached to C, N and O atoms were fixed geometrically and treated as riding, with C—H = 0.93 Å, N—H = 0.86 Å and O—H = 0.82 Å and with *U*_iso_(H) = 1.2Ueq(C or N) and *U*_iso_(H) = 1.5Ueq(O)

### Crystal data

**Table:**

2C_5_H_6_N_3_O_2_^+^·H_2_O_7_P_2_^2^^−^	*F*(000) = 936
*M**_r_* = 456.21	*D*_x_ = 1.707 Mg m^−^^3^
Orthorhombic, *P**c**a*2_1_	Mo *K*α radiation, λ = 0.71073 Å
Hall symbol: P 2c -2ac	Cell parameters from 25 reflections
*a* = 34.250 (5) Å	θ = 10–12°
*b* = 5.763 (2) Å	µ = 0.32 mm^−^^1^
*c* = 8.991 (3) Å	*T* = 298 K
*V* = 1774.8 (9) Å^3^	Prism, yellow
*Z* = 4	0.29 × 0.25 × 0.19 mm

### Data collection

**Table:**

Enraf–Nonius CAD-4 diffractometer	*R*_int_ = 0.008
Radiation source: fine-focus sealed tube	θ_max_ = 30.0°, θ_min_ = 3.3°
graphite	*h* = −48→0
non–profiled ω scans	*k* = 0→8
2726 measured reflections	*l* = −12→0
2724 independent reflections	2 standard reflections every 120 min
2279 reflections with *I* > 2σ(*I*)	intensity decay: 3%

### Refinement

**Table:**

Refinement on *F*^2^	Hydrogen site location: inferred from neighbouring sites
Least-squares matrix: full	H-atom parameters constrained
*R*[*F*^2^ > 2σ(*F*^2^)] = 0.033	*w* = 1/[σ^2^(*F*_o_^2^) + (0.0391*P*)^2^ + 0.7964*P*] where *P* = (*F*_o_^2^ + 2*F*_c_^2^)/3
*wR*(*F*^2^) = 0.091	(Δ/σ)_max_ = 0.001
*S* = 1.09	Δρ_max_ = 0.53 e Å^−^^3^
2724 reflections	Δρ_min_ = −0.28 e Å^−^^3^
265 parameters	Extinction correction: *SHELXL97* (Sheldrick, 2008), Fc^*^=kFc[1+0.001xFc^2^λ^3^/sin(2θ)]^-1/4^
1 restraint	Extinction coefficient: 0.0192 (12)
Primary atom site location: structure-invariant direct methods	Absolute structure: Flack (1983)
Secondary atom site location: difference Fourier map	Flack parameter: −0.15 (11)

### Special details

**Table:**

Geometry. All e.s.d.'s (except the e.s.d. in the dihedral angle between two l.s. planes) are estimated using the full covariance matrix. The cell e.s.d.'s are taken into account individually in the estimation of e.s.d.'s in distances, angles and torsion angles; correlations between e.s.d.'s in cell parameters are only used when they are defined by crystal symmetry. An approximate (isotropic) treatment of cell e.s.d.'s is used for estimating e.s.d.'s involving l.s. planes.
Refinement. Refinement of *F*^2^ against ALL reflections. The weighted *R*-factor *wR* and goodness of fit *S* are based on *F*^2^, conventional *R*-factors *R* are based on *F*, with *F* set to zero for negative *F*^2^. The threshold expression of *F*^2^ > σ(*F*^2^) is used only for calculating *R*-factors(gt) *etc*. and is not relevant to the choice of reflections for refinement. *R*-factors based on *F*^2^ are statistically about twice as large as those based on *F*, and *R*- factors based on ALL data will be even larger.

### Fractional atomic coordinates and isotropic or equivalent isotropic displacement parameters (Å^2^)

**Table:**

	*x*	*y*	*z*	*U*_iso_*/*U*_eq_	
P1	0.05181 (2)	0.76331 (12)	0.64677 (9)	0.02939 (15)	
P2	0.06740 (2)	0.49228 (13)	0.38188 (9)	0.02940 (15)	
O1	0.02869 (6)	0.6068 (4)	0.7473 (3)	0.0388 (5)	
O2	0.03160 (6)	1.0043 (4)	0.6468 (3)	0.0380 (5)	
H2	0.0432	1.0916	0.5898	0.057*	
O3	0.09419 (6)	0.7686 (4)	0.6795 (3)	0.0406 (5)	
O4	0.04503 (6)	0.6839 (4)	0.4767 (2)	0.0349 (4)	
O5	0.06725 (7)	0.2687 (4)	0.4645 (3)	0.0442 (6)	
O6	0.10798 (6)	0.5779 (4)	0.3433 (3)	0.0381 (5)	
O7	0.04355 (6)	0.4892 (4)	0.2361 (2)	0.0367 (5)	
H7	0.0207	0.4592	0.2548	0.055*	
O8	0.17007 (11)	0.0987 (8)	1.2413 (5)	0.0996 (14)	
O9	0.17848 (8)	0.4036 (7)	1.1115 (4)	0.0796 (12)	
O10	0.17870 (9)	−0.0306 (6)	0.8224 (4)	0.0689 (9)	
O11	0.23687 (9)	0.0861 (7)	0.8703 (5)	0.0899 (12)	
N1	0.07030 (7)	0.3161 (5)	0.9226 (3)	0.0334 (5)	
H1	0.0606	0.4078	0.8567	0.040*	
N2	0.12502 (8)	0.5449 (5)	0.9194 (3)	0.0445 (7)	
H2A	0.1139	0.6299	0.8532	0.053*	
H2B	0.1481	0.5788	0.9499	0.053*	
N3	0.15874 (9)	0.2373 (7)	1.1479 (5)	0.0578 (9)	
N4	0.16725 (8)	0.4967 (5)	0.5130 (4)	0.0396 (6)	
H4	0.1465	0.5191	0.4608	0.048*	
N5	0.13779 (8)	0.1725 (6)	0.6059 (4)	0.0522 (8)	
H5A	0.1184	0.1998	0.5479	0.063*	
H5B	0.1374	0.0536	0.6637	0.063*	
N6	0.20652 (10)	0.1031 (6)	0.8028 (4)	0.0548 (8)	
C1	0.04851 (9)	0.1383 (5)	0.9672 (4)	0.0374 (6)	
H1A	0.0236	0.1196	0.9281	0.045*	
C2	0.06201 (11)	−0.0169 (6)	1.0689 (4)	0.0450 (8)	
H2C	0.0469	−0.1431	1.0978	0.054*	
C3	0.09860 (10)	0.0180 (6)	1.1278 (4)	0.0453 (8)	
H3	0.1084	−0.0843	1.1986	0.054*	
C4	0.12081 (9)	0.2041 (6)	1.0822 (4)	0.0404 (7)	
C5	0.10672 (8)	0.3631 (5)	0.9740 (4)	0.0350 (6)	
C6	0.19675 (10)	0.6453 (6)	0.4979 (5)	0.0470 (8)	
H6	0.1943	0.7678	0.4312	0.056*	
C7	0.23056 (10)	0.6223 (7)	0.5778 (5)	0.0550 (10)	
H7C	0.2512	0.7255	0.5652	0.066*	
C8	0.23302 (9)	0.4406 (7)	0.6779 (4)	0.0511 (9)	
H8	0.2554	0.4221	0.7352	0.061*	
C9	0.20255 (9)	0.2872 (6)	0.6933 (4)	0.0417 (7)	
C10	0.16813 (9)	0.3125 (6)	0.6061 (4)	0.0389 (7)	

### Atomic displacement parameters (Å^2^)

**Table:**

	*U*^11^	*U*^22^	*U*^33^	*U*^12^	*U*^13^	*U*^23^
P1	0.0326 (3)	0.0309 (3)	0.0247 (3)	−0.0046 (3)	−0.0008 (3)	0.0044 (3)
P2	0.0323 (3)	0.0269 (3)	0.0291 (3)	−0.0007 (3)	0.0004 (3)	0.0045 (3)
O1	0.0377 (10)	0.0429 (12)	0.0357 (11)	−0.0058 (9)	−0.0001 (9)	0.0130 (10)
O2	0.0473 (11)	0.0334 (10)	0.0333 (11)	−0.0004 (9)	0.0047 (10)	0.0004 (11)
O3	0.0373 (10)	0.0474 (12)	0.0371 (13)	−0.0090 (10)	−0.0036 (9)	0.0097 (10)
O4	0.0379 (10)	0.0374 (10)	0.0293 (10)	0.0054 (9)	−0.0045 (9)	0.0000 (9)
O5	0.0461 (13)	0.0321 (11)	0.0544 (15)	−0.0020 (9)	0.0013 (11)	0.0162 (11)
O6	0.0336 (10)	0.0412 (11)	0.0394 (12)	−0.0049 (9)	0.0003 (9)	0.0102 (10)
O7	0.0357 (10)	0.0457 (12)	0.0288 (10)	−0.0050 (10)	−0.0004 (9)	−0.0028 (10)
O8	0.072 (2)	0.126 (3)	0.101 (3)	0.007 (2)	−0.035 (2)	0.058 (3)
O9	0.0414 (14)	0.108 (3)	0.089 (3)	−0.0142 (16)	−0.0172 (16)	0.035 (2)
O10	0.0647 (18)	0.073 (2)	0.069 (2)	−0.0055 (16)	−0.0033 (15)	0.0349 (18)
O11	0.0635 (19)	0.114 (3)	0.092 (3)	0.0031 (19)	−0.025 (2)	0.045 (3)
N1	0.0393 (13)	0.0353 (13)	0.0257 (11)	0.0034 (10)	−0.0001 (9)	0.0036 (10)
N2	0.0426 (14)	0.0462 (16)	0.0447 (17)	−0.0056 (12)	−0.0057 (12)	0.0137 (13)
N3	0.0423 (15)	0.079 (2)	0.0518 (17)	0.0116 (17)	−0.0067 (16)	0.018 (2)
N4	0.0325 (11)	0.0423 (14)	0.0440 (15)	0.0013 (11)	−0.0029 (11)	0.0083 (13)
N5	0.0423 (15)	0.0524 (17)	0.062 (2)	−0.0119 (13)	−0.0101 (15)	0.0222 (16)
N6	0.0492 (16)	0.066 (2)	0.0494 (18)	0.0083 (16)	0.0005 (15)	0.0154 (17)
C1	0.0393 (15)	0.0418 (16)	0.0309 (14)	−0.0026 (13)	0.0037 (13)	−0.0027 (14)
C2	0.0549 (19)	0.0353 (16)	0.0447 (18)	−0.0021 (15)	0.0087 (16)	0.0062 (15)
C3	0.0535 (17)	0.0414 (16)	0.0411 (19)	0.0132 (14)	0.0058 (15)	0.0119 (16)
C4	0.0373 (15)	0.0488 (18)	0.0350 (16)	0.0079 (13)	−0.0004 (13)	0.0054 (15)
C5	0.0372 (14)	0.0390 (16)	0.0288 (13)	0.0054 (12)	0.0018 (12)	0.0035 (13)
C6	0.0419 (17)	0.0463 (18)	0.053 (2)	−0.0023 (14)	0.0041 (15)	0.0086 (17)
C7	0.0374 (17)	0.061 (2)	0.066 (3)	−0.0102 (16)	0.0042 (18)	0.008 (2)
C8	0.0303 (14)	0.072 (2)	0.051 (2)	0.0006 (16)	−0.0028 (15)	0.0070 (19)
C9	0.0329 (13)	0.0520 (18)	0.0404 (15)	0.0050 (13)	0.0030 (12)	0.0089 (16)
C10	0.0345 (14)	0.0408 (16)	0.0412 (16)	0.0024 (13)	0.0042 (13)	0.0041 (13)

### Geometric parameters (Å, °)

**Table:**

P1—O3	1.481 (2)	N4—C6	1.331 (4)
P1—O1	1.503 (2)	N4—C10	1.353 (4)
P1—O2	1.552 (2)	N4—H4	0.8600
P1—O4	1.613 (2)	N5—C10	1.316 (4)
P2—O5	1.487 (2)	N5—H5A	0.8600
P2—O6	1.515 (2)	N5—H5B	0.8600
P2—O7	1.545 (2)	N6—C9	1.454 (5)
P2—O4	1.592 (2)	C1—C2	1.360 (5)
O2—H2	0.8200	C1—H1A	0.9300
O7—H7	0.8200	C2—C3	1.375 (5)
O8—N3	1.222 (5)	C2—H2C	0.9300
O9—N3	1.217 (5)	C3—C4	1.377 (5)
O10—N6	1.238 (4)	C3—H3	0.9300
O11—N6	1.208 (4)	C4—C5	1.420 (4)
N1—C1	1.330 (4)	C6—C7	1.369 (5)
N1—C5	1.358 (4)	C6—H6	0.9300
N1—H1	0.8600	C7—C8	1.383 (5)
N2—C5	1.316 (4)	C7—H7C	0.9300
N2—H2A	0.8600	C8—C9	1.375 (5)
N2—H2B	0.8600	C8—H8	0.9300
N3—C4	1.440 (4)	C9—C10	1.423 (5)
			
O3—P1—O1	114.15 (13)	O10—N6—C9	118.6 (3)
O3—P1—O2	114.74 (13)	N1—C1—C2	121.2 (3)
O1—P1—O2	107.60 (13)	N1—C1—H1A	119.4
O3—P1—O4	109.59 (13)	C2—C1—H1A	119.4
O1—P1—O4	108.93 (14)	C1—C2—C3	118.2 (3)
O2—P1—O4	100.93 (13)	C1—C2—H2C	120.9
O5—P2—O6	113.55 (13)	C3—C2—H2C	120.9
O5—P2—O7	114.34 (15)	C2—C3—C4	120.2 (3)
O6—P2—O7	107.14 (13)	C2—C3—H3	119.9
O5—P2—O4	109.41 (14)	C4—C3—H3	119.9
O6—P2—O4	109.76 (13)	C3—C4—C5	121.2 (3)
O7—P2—O4	101.99 (12)	C3—C4—N3	118.7 (3)
P1—O2—H2	109.5	C5—C4—N3	120.1 (3)
P2—O4—P1	129.43 (14)	N2—C5—N1	118.0 (3)
P2—O7—H7	109.5	N2—C5—C4	127.4 (3)
C1—N1—C5	124.5 (3)	N1—C5—C4	114.6 (3)
C1—N1—H1	117.7	N4—C6—C7	121.7 (4)
C5—N1—H1	117.7	N4—C6—H6	119.1
C5—N2—H2A	120.0	C7—C6—H6	119.1
C5—N2—H2B	120.0	C6—C7—C8	117.8 (3)
H2A—N2—H2B	120.0	C6—C7—H7C	121.1
O9—N3—O8	121.5 (4)	C8—C7—H7C	121.1
O9—N3—C4	119.7 (3)	C9—C8—C7	120.4 (3)
O8—N3—C4	118.8 (4)	C9—C8—H8	119.8
C6—N4—C10	123.4 (3)	C7—C8—H8	119.8
C6—N4—H4	118.3	C8—C9—C10	120.5 (3)
C10—N4—H4	118.3	C8—C9—N6	117.8 (3)
C10—N5—H5A	120.0	C10—C9—N6	121.7 (3)
C10—N5—H5B	120.0	N5—C10—N4	117.6 (3)
H5A—N5—H5B	120.0	N5—C10—C9	126.3 (3)
O11—N6—O10	122.7 (4)	N4—C10—C9	116.1 (3)
O11—N6—C9	118.7 (3)		
			
O5—P2—O4—P1	53.0 (2)	N3—C4—C5—N2	1.9 (6)
O6—P2—O4—P1	−72.2 (2)	C3—C4—C5—N1	0.4 (5)
O7—P2—O4—P1	174.45 (18)	N3—C4—C5—N1	−178.7 (3)
O3—P1—O4—P2	39.2 (2)	C10—N4—C6—C7	−0.5 (6)
O1—P1—O4—P2	−86.3 (2)	N4—C6—C7—C8	−1.1 (6)
O2—P1—O4—P2	160.61 (18)	C6—C7—C8—C9	1.2 (6)
C5—N1—C1—C2	−1.3 (5)	C7—C8—C9—C10	0.2 (6)
N1—C1—C2—C3	1.6 (5)	C7—C8—C9—N6	−178.6 (4)
C1—C2—C3—C4	−0.9 (5)	O11—N6—C9—C8	−3.4 (6)
C2—C3—C4—C5	−0.1 (5)	O10—N6—C9—C8	177.0 (4)
C2—C3—C4—N3	179.0 (4)	O11—N6—C9—C10	177.8 (4)
O9—N3—C4—C3	−178.4 (4)	O10—N6—C9—C10	−1.8 (5)
O8—N3—C4—C3	0.5 (6)	C6—N4—C10—N5	−176.9 (4)
O9—N3—C4—C5	0.7 (6)	C6—N4—C10—C9	1.9 (5)
O8—N3—C4—C5	179.6 (4)	C8—C9—C10—N5	177.0 (4)
C1—N1—C5—N2	179.7 (3)	N6—C9—C10—N5	−4.2 (6)
C1—N1—C5—C4	0.2 (4)	C8—C9—C10—N4	−1.7 (5)
C3—C4—C5—N2	−179.0 (3)	N6—C9—C10—N4	177.1 (3)

### Hydrogen-bond geometry (Å, °)

**Table:**

*D*—H···*A*	*D*—H	H···*A*	*D*···*A*	*D*—H···*A*
O2—H2···O5^i^	0.82	1.73	2.549 (3)	178
O7—H7···O1^ii^	0.82	1.73	2.537 (3)	166
N1—H1···O1	0.86	1.87	2.706 (3)	165
N2—H2A···O3	0.86	1.88	2.726 (4)	168
N2—H2B···O9	0.86	2.05	2.645 (4)	126
N4—H4···O6	0.86	1.72	2.582 (4)	174
N5—H5A···O5	0.86	1.95	2.786 (4)	165
N5—H5B···O10	0.86	2.07	2.669 (5)	126
N5—H5B···O3^iii^	0.86	2.22	2.843 (4)	130
C2—H2C···O7^iv^	0.93	2.46	3.280 (4)	147
C3—H3···O6^iv^	0.93	2.34	3.208 (4)	155
C8—H8···O9^v^	0.93	2.52	3.097 (4)	120

Symmetry codes: (i) *x*, *y*+1, *z*; (ii) −*x*, −*y*+1, *z*−1/2; (iii) *x*, *y*−1, *z*; (iv) *x*, *y*−1, *z*+1; (v) −*x*+1/2, *y*, *z*−1/2.
